# Effect of a subanesthetic dose of esketamine combined with propofol on postoperative fatigue syndrome in patients undergoing gastroenterological endoscopy under anaesthesia: A retrospective study

**DOI:** 10.12669/pjms.40.8.9883

**Published:** 2024-09

**Authors:** Guofang Fei, Wei Yan, Yuehua Gan

**Affiliations:** 1 Guofang Fei Department of Anesthesiology, Huzhou Maternity & Child Health Care Hospital, 2 East Street, Huzhou City, Zhejiang Province, P.R. China; 2 Wei Yan Department of Anesthesiology, Huzhou Maternity & Child Health Care Hospital, 2 East Street, Huzhou City, Zhejiang Province, P.R. China; 3 Yuehua Gan Department of Anesthesiology, Huzhou Maternity & Child Health Care Hospital, 2 East Street, Huzhou City, Zhejiang Province, P.R. China

**Keywords:** Esketamine, Subanesthetic dose, Gastroenterological endoscopy, Postoperative fatigue syndrome

## Abstract

**Objective:**

To assess the effect of subanesthetic dose of esketamine in combination with propofol on the incidence of postoperative fatigue syndrome (POFS) in patients who underwent gastroenterological endoscopy under anaesthesia.

**Methods:**

Clinical data of 160 patients who underwent gastroenterological endoscopy under anaesthesia in Huzhou Maternity & Child Health Care Hospital from January to December 2022, ASA Grade- I and II, were retrospectively selected. According to the records, patients were grouped based on the administered anesthetic. Patients who received 0.2 mg/kg of esketamine and 2~2.5mg/kg of propofol comprised Group-E, and patients who were administered one μg/kg of fentanyl and 2 - 2.5mg/kg of propofol comprised Group-F. Mean arterial pressure (MAP), oxygen saturation (SpO_2_) and heart rate (HR) were recorded before the operation (T_0_), after anesthesia (T_1_), three minutes after the gastroscope was inserted (T_2_), five minutes after the colonoscope was inserted (T_3_) and at the end of the operation (T_4_). Operating time, recovery time, propofol dosage and incidence of adverse reactions in the two groups were recorded. The Christensen scores and the incidence of POFS of all patients on Day-I before operation and 1st, 3rd, and 5th days after the operation were recorded.

**Results:**

Compared with T_0_, MAP, SpO_2_ and HR in both groups of patients decreased at T_1_, T_2_, T_3_ and T_4_ (*P*<0.05). MAP, SpO_2_ and HR of patients in Group-E were significantly higher compared to Group-F at T_1_, T_2_, T_3_ and T_4_ (*P*<0.05). Compared with Group-F, the recovery time, intraoperative bradycardia and respiratory depression in Group-E were significantly lower (*P*<0.05), and Christensen scores and the incidence of POFS decreased significantly on the 1st, 3rd, and 5th day after the operation (*P*<0.05).

**Conclusion:**

Subanesthetic dose of esketamine combined with propofol can reduce POFS and postoperative adverse reactions in patients undergoing gastroenterological endoscopy.

## INTRODUCTION

Postoperative fatigue syndrome (POFS) is a group of symptoms that occur in patients who undergone surgery and is characterized by a feeling of fatigue, drowsiness, and inattention. POFS may impact postoperative recovery of patients to varying degrees and prolong their hospital stay.^1^ Since anesthesia is considered an independent risk factor for the development of POFS,^2^ the risk of POFS after gastroenterological endoscopy procedures that are performed under anesthesia is significantly higher than that of plain gastroenterological endoscopy.^3^ Esketamine is an antagonist of N-methyl-D-aspartate (NMDA) receptor, a new type of intravenous anesthetic with concomitant sedative-analgesic effects, and rapid onset.^4^

Current studies show that the subanesthetic dose of esketamine, which has circulatory excitatory effects and does not inhibit respiration, provides satisfactory anesthesia and reduces the incidence of adverse effects when used for gastroenterological endoscopy.^5^-^7^ However, there are few inconclusive reports on the effects of esketamine on postoperative fatigue syndrome in patients.^8^–^10^ While some studies have reported that esketamine during the perioperative phase is beneficial for overall postoperative recovery,^11^–^13^ others find no such meaningful association.^14^ Since POFS negatively affects recovery process, prolonges hospital stays and markedly impacts quality of life of patients, assessing the effect of esketamine on the occurrence of POFS is crucial for improving postoperative outcomes of patients undergoing gastroenterological endoscopy. In this study, we retrospectively assessed the effect of subanesthetic dose of esketamine on postoperative fatigue syndrome in this group of patients to provide clinical reference.

## METHODS

The retrospective study included medical records of 160 patients who underwent gastroenterological endoscopy under anaesthesia in Huzhou Maternity & Child Health Care Hospital from January to December 2022. All patients had the American Society of Anesthesiologists (*ASA*) physical status *classification* I-II, were aged 18-70 years, and weighted 45-85 kg. Based on the type of anaesthesia during the procedure, patients were retrospectively divided into esketamine + propofol group (Group-E) and fentanyl + propofol group (Group-F), 80 patients each.

### Inclusion criteria:

- Undergoing combined gastroenterological endoscopy, ability to fully understand and participate in postoperative scoring.

### Exclusion criteria:

- Patients with severe heart, lungs, liver and kidneys conditions, history of psychiatric illness, difficult airway, or history of allergy to anesthesia drugs.

### Ethical Approval:

Ethics Committee of the hospital reviewed and approved the study, and informed consent was obtained from the patients. Ethical approval number 2023-J-079, date: August 23, 2023.

### Assessment and follow up:

Before receiving anesthesia, patients were required to attend the clinic for a pre-anesthetic assessment. For the anesthetic assessment, a 1-day pre-operative Christensen score was performed, and the patients’ Christensen scores were followed up by telephone by the anesthesiologist on days one, three and five postoperatively and the results were recorded.

All patients underwent routine preoperative gastrointestinal preparation, and 10 ml of lidocaine gel paste was administered orally 15 min before the examination. After entering the examination room, patients were placed in the left lateral position, oxygen was administered by mask (oxygen flow rate of five L/minutes), intravenous access was established, and routine electrocardiographic monitoring was performed to monitor noninvasive blood pressure (NIBP), pulse oximetry (SpO_2_), and heart rate (HR).

### Anesthesia in Group-E patients:

Before the start of gastroenterological endoscopy, patients were given a single injection of esketamine 0.2mg/kg, followed by a slow infusion of propofol 2-2.5mg/kg.

### Anesthesia in Group-F patients:

patients were given a single injection of fentanyl 1μg/kg, followed by a slow injection of propofol 2-2.5mg/kg, and the examination started after the patients’ eyelash reflex disappeared.

During the procedure, in case of obvious body movement, propofol 1-2 mg/kg was added. If heart rate was <50 beats/min, 0.5 mg atropine was injected. If systolic blood pressure (SBP) decreased by ≥30% of the preoperative period or <90 mmHg, ephedrine 10-15 mg was injected. In cases of respiratory inhibition, appropriate treatments were undertaken, such as supporting the lower jaw, artificial ventilation, etc. All anesthetic operations were performed by the same anesthetist, gastroenterological endoscopy was performed by the same endoscopist, and all data collection was performed by another anesthetist who was unaware of the grouping.

### Observation indicators:

Mean arterial pressure (MAP), pulse oximetry (SpO_2_) and heart rate (HR) values were collected preoperatively (T_0_), immediately after anesthesia (T_1_), three minutes after gastroscope insertion (T_2_), five minutes after enteroscope insertion (T_3_), and at the end of the procedure (T_4_) in both groups. The time of gastroenterological endoscopy, the time of anesthesia awakening (the time from the end of the colonoscopy to the patient’s eyes opening), the dosage of propofol and the incidence of side effects were recorded in both groups.

Christensen Fatigue Score and the incidence of POFS were recorded at Day-I preoperatively, first, third and fifth day postoperatively in both groups. Christensen fatigue score^15^ assesses the severity of POFS. Score of 1-2 is indicative of normal condition; fatigue during excessive activity, normal sleep receives a score 3-4; score of 5-6 is given to patients who are able to maintain normal life, can occasionally engage in slightly forceful activities; patients who can only engage in simple activities, feel strained when going up the stairs, walking, and need to sleep receive a score of 7-8; patients who are unable to carry out daily activities, and in urgent need of sleep get score 9-10. Christensen score ≥ 6 indicates clinically visible and more obvious POFS.^16^ In this study, the incidence of POFS was the main observation index (Supplementary Fig.1).^15^

**Supplementry Fig.1:**
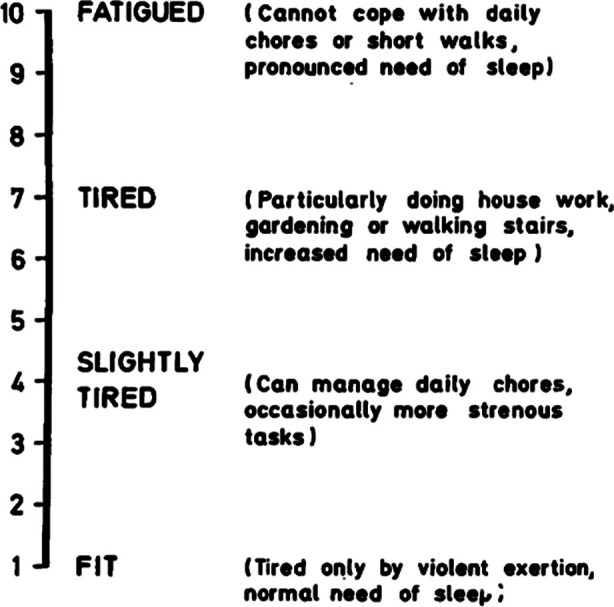
Self-evaluation of fatigue using Christensen Fatigue Score.^15^

### Statistical analysis:

SPSS26.0 statistical software was used to analyze the data. Normally distributed measurements were presented as mean ± standard deviation (*χ̅*±*S*), and comparison between groups was done by the t-test. Count data were expressed as cases (%), analyzed by χ^2^ test or Fisher’s exact probability method. P < 0.05 indicated statistical significance.

## RESULTS

Medical data of 160 patients were included in this study, with no statistically significant difference in age, male/female ratio, ASA classification, BMI, and duration of surgery between groups E and F (Table-I). An intra-group comparison showed that MAP, SpO_2_, and HR at T_1_, T_2_, T_3_, and T_4_ were significantly lower than those at T_0_ in both groups (P < 0.05). An inter-group comparison demonstrated that MAP, SpO_2_, and HR at T_1_, T_2_, T_3_, and T_4_ were significantly higher in patients in Group-E compared to Group-F at the same time points (P < 0.05) (Table-II).

**Table-I:** Comparison of the general information of patients in Group-E and F (*χ̅*±*S*).

Group	Case number (cases)	Age (years)	Sex ratio (male/female)	ASA Classification (I/II)	BMI (kg/m^2)^	Surgical time (min)
Group-E	80	48.4±10.5	30/50	45/35	23.7±2.2	35.3±7.6
Group-F	80	47.6±11.3	27/53	48/32	24.1±2.5	34.7±6.8

**Table-II:** Comparison of vital signs at various time points between the two groups of patients (*χ̅*±*S*).

Group	Events	T_0_	T_1_	T_2_	T_3_	T_4_
Group-E	MAP (mmHg)	85.4±7.4	78.6±6.3^*#^	74.7±6.1^*#^	72.8±5.3^*#^	73.3±4.6^*#^
Group-F		84.6±6.7	65.7±7.5^*^	65.6±5.6^*^	65.7±5.5^*^	66.8±4.1^*^
Group-E	SpO_2_ (%)	99.0±0.4	98.3±0.6^*#^	98.2±0.1^*#^	98.8±0.2^*#^	98.8±0.1^*#^
Group-F		99.0±0.6	97.1±0.3^*^	97.7±0.3^*^	98.4±0.2^*^	98.6±0.3^*^
Group-E	HR (times/min)	87.6±10.5	83.3±7.6^*#^	83.8±6.4^*#^	83.5±4.6^*#^	84.0±4.3^*#^
Group-F		88.4±10.8	80.5±6.0^*^	80.1±6.2^*^	80.2±5.9^*^	81.5±6.1^*^

***Note:*** Compared with T0,

* P<0.05; compared with Group-F, #P<0.05.

Compared with Group-F, the incidence of anesthesia awakening time, intraoperative bradycardia and respiratory depression were significantly lower in Group-E patients (P < 0.05). Propofol dosage and the incidence of other adverse reactions was comparable in the two groups (P > 0.05), (Table-III). The Christensen scores of patients in Group-E were significantly lower at first, third and fifth day postoperatively than in Group-F (P < 0.05). The incidence of POFS at first, third and fifth day postoperatively was significantly lower in Group-E compared with Group-F (P < 0.05), (Table-IV).

**Table-III:** Comparison of anesthesia and incidence of adverse reactions between the two groups of patients.

Group	Wake-up time (min)	Propofol (mg)	Bradycardia (cases, %)	Respiratory depression (cases, %)	Injection pain (cases, %)	Nausea and vomiting (cases, %)
Group-E	3.6±1.2^#^	251.9±25.6	3(3.75%)^#^	3(3.75%)^#^	6(7.5%)	4(5%)
Group-F	4.2±1.3	248.6±24.7	11(13.75%)	15(18.75%)	10(12.5%)	6(7.5%)

***Note:*** #P<0.05 compared to Group-F.

**Table-IV:** Comparison of postoperative Christensen score and occurrence of POFS between the two groups of patients.

Group	Events	1 day before surgery	1 day after surgery	3 days after surgery	5 days after surgery
Group-E	Christensen score	1.9±0.2	4.2±1.5^*#^	3.9±1.0^*#^	3.3±0.6^*#^
Group-F		1.9±0.3	6.9±1.7^*^	5.6±1.5^*^	4.8±1.1^*^
Group-E	POFS incidence		15(18.75%)^#^	12(15%)^#^	6(7.5%)^#^
Group-F			30(37.5%)	28(35%)	17(22.5%)

***Note:*** Compared with one day before surgery,

* P<0.05; compared with Group-F, #P<0.05.

## DISCUSSION

This retrospective study showed that subanesthetic dose of esketamine in combination with propofol can reduce the incidence of POFS and postoperative adverse reactions, such as intraoperative bradycardia and respiratory depression, in patients undergoing gastroenterological endoscopy. The incidence of POFS after surgical procedures ranges from 34% to 87%.^17^,^18^ While most previous studies of POFS have focused on major surgical procedures, it is not uncommon for patients to develop POFS after outpatient procedures such as gastroenterological endoscopy.^19^

The etiology of POFS is complex and may result from a combination of physiological and psychological factors. Surgical trauma, anesthesia factors, nutritional status, inflammation levels, negative emotions and social support may all influence the occurrence and development of POFS in surgical patients.^20^,^21^ Our results showed that a subanesthetic dose of esketamine together with propofol for gastroenterological endoscopy was effective in reducing patients’ postoperative Christensen scores and the incidence of POFS.

The Christensen score is an internationally recognized fatigue scale that provides a good description of the subjective physical and psychological feelings of patients after the surgery. Gastroenterological endoscopy is an invasive diagnostic and therapeutic procedure regardless of the use of anesthesia, and affects the patient’s psychological state to varying degrees, potentially leading to post-procedural fatigue. Esketamine has significant antidepressant effects that continue for about seven days after discontinuation of the drug.^22^ It has been shown that esketamine-combined anesthesia can reduce the incidence of postoperative psychological distress in patients.^23^

The psychological aspects of the pharmacological effects of esketamine may explain our observation of significantly lower Christensen scores in Group-E patients compared to patients in Group-F at one, three and five days postoperatively. Physiologically, esketamine exerts analgesic effects by acting on NMDA and opioid μ receptors.^24^ Our study did not detect significant difference in the amount of propofol used to complete gastroenterological endoscopy in patients in Groups E and F. This indicates that the subanesthetic dose of esketamine is not inferior to fentanyl for adjuvant analgesia. Additionally, it excludes the potential effect of the propofol dosage on POFS. We may hypothesize that the observed decrease in the incidence of POFS in Group-E may be due to the fact that subanesthetic dose of esketamine does not inhibit respiration.

Additionally, esketamine also has a sympathetic excitatory effect that is able to neutralize the cardiovascular inhibitory effect of propofol.^25^,^26^ Previous studies showed that excessive hemodynamic fluctuations are a risk factor for the occurrence of POFS.^27^ Our results demonstrated that Group-E patients had relatively stable intraoperative hemodynamics, which may further explain lower incidence of POFS in patients who were administered esketamine. Furthermore, animal experiments have confirmed that inflammatory factors may stimulate tryptophan metabolism and lead to POFS through the NMDA receptor pathway.^28^ Esketamine is an NMDA receptor antagonist, and can reduce the incidence of POFS by blocking this pathway. Additionally, esketamine also anti-inflammatory and central neuroprotective effects,^29^ all of which may contribute to lower rates of POFS, associated with esketamine use. In our study, the awakening time of patients in Group-E was significantly lower than in Group-F, which is consistent with the results of Wang et al.’s study.^30^

### Limitations:

It is a retrospective study with a small sample size, which prevented randomization, which may impact the generalizability of our results. Additionally, this study used esketamine or fentanyl combined with propofol for gastroscopy anesthesia. However, opioid drugs have been proven to affect postoperative recovery in patients. Therefore, future studies that exclude opioid drugs evaluate other methods of anaesthesia in the context of POFS are needed. Further double-blinded, randomized and placebo-controlled trials on the basis of this study are needed.

## CONCLUSION

Our retrospective study demonstrated that sub-anesthetic dose of esketamine combined with propofol for gastroenterological endoscopy can effectively lower the occurrence of POFS in patients and reduce postoperative adverse reactions. This method of anesthesia, therefore, may be clinically promoted for painless gastroenterological endoscopy.

### Authors’ Contributions:

**GF:** Conceived and designed the study.

**GF**, **WY** and **YG:** Collected the data and performed the analysis.

**GF:** Was involved in the writing of the manuscript and is responsible for the integrity of the study.

All authors have read and approved the final manuscript.
